# Full genome comparison and characterization of avian H10 viruses with different pathogenicity in Mink (*Mustela vison*) reveals genetic and functional differences in the non-structural gene

**DOI:** 10.1186/1743-422X-7-145

**Published:** 2010-06-30

**Authors:** Siamak Zohari, Giorgi Metreveli, István Kiss, Sándor Belák, Mikael Berg

**Affiliations:** 1 Swedish University of Agricultural Sciences (SLU), Department of Biomedical Sciences and Public Health, Section of Virology, SLU, Ulls väg 2B, SE-751 89 Uppsala, Sweden; 2 National Veterinary Institute, SVA, Unit for Virology, Immunobiology, and Parasitology, Ulls väg 2B, SE-751 89 Uppsala, Sweden

## Abstract

**Background:**

The unique property of some avian H10 viruses, particularly the ability to cause severe disease in mink without prior adaptation, enabled our study. Coupled with previous experimental data and genetic characterization here we tried to investigate the possible influence of different genes on the virulence of these H10 avian influenza viruses in mink.

**Results:**

Phylogenetic analysis revealed a close relationship between the viruses studied. Our study also showed that there are no genetic differences in receptor specificity or the cleavability of the haemagglutinin proteins of these viruses regardless of whether they are of low or high pathogenicity in mink.

In poly I:C stimulated mink lung cells the NS1 protein of influenza A virus showing high pathogenicity in mink down regulated the type I interferon promoter activity to a greater extent than the NS1 protein of the virus showing low pathogenicity in mink.

**Conclusions:**

Differences in pathogenicity and virulence in mink between these strains could be related to clear amino acid differences in the non structural 1 (NS1) protein. The NS gene of mink/84 appears to have contributed to the virulence of the virus in mink by helping the virus evade the innate immune responses.

## Background

The outbreak of severe respiratory disease in mink (*Mustela vison) *in 1984 was linked to an avian influenza virus of subtype H10N4. At the time this was the first known outbreak of avian influenza A virus infection in a terrestrial mammalian species [1,2]. The only possible explanation was that birds carrying the virus transmitted it via their faeces to the mink. At the time, this was one of the very first cases of direct transmission of avian influenza virus to a terrestrial mammalian species [1].

Only a few months after the outbreak in Swedish mink, some viruses of the H10N4 subtype were isolated from domestic and wild birds in Great Britain [3]. Rather crude full-genomic comparison by oligonucleotide (ON) mapping [4] and sequence analysis of the HA [5] and NP genes [6] were conducted. The ON mapping showed a close genomic relationship between the mink isolate (A/Mink/Sweden/3900/84) and the concomitant avian H10N4 viruses from fowl (A/fowl/Hampshire/378/85) and mallard (A/mallard/Gloucestershire/374/85) respectively, and a weaker genomic relationship with the H10 prototype [7] virus (A/chicken/Germany/N/49) [4].

Experimental infection of mink (*Mustela vison) *was initially used to link the isolated influenza virus to the clinical symptoms and pathological lesions observed in the field outbreak. In a later study, mink were infected intranasally with mink/84, mallard/85, fowl/85, or chicken/49 to compare clinical symptoms, antibody response, and possible in-contact transmission [4].

Experimental aerosol infections of mink, using mink/84 or chicken/49, were then used to compare in more detail the pathogenesis of the two virus infections [8,9]. Following intranasal infection of the mink, all three H10N4 isolates, i.e. mink/84, mallard/85 and fowl/85, showed similar clinical symptoms, causing respiratory disease, interstitial pneumonia and specific antibody production. All three H10N4 isolates were transmitted via contact infection. Chicken/49 did not cause clinical disease or contact infection, but induced antibody production and mild lung lesions [8].

Further comparison between mink/84 and chicken/49 revealed that the infections progressed with similar patterns over the first 24 hours post infection but from 48 hours post infection obvious differences were recorded. In mink infected with chicken/49 no signs of disease were observed, while the mink infected with mink/84 showed severe signs of respiratory disease, with inflammatory lesions spreading throughout the lung and viral antigen present in substantial numbers of cells in the lung, nasal mucosa, and trachea. The chicken/49 and mink/84 virus have also been shown to differ in their ability to induce interferon (IFN) production in mink lung cells [8-10].

In an effort to better understand the mechanism behind the virulence of influenza A viruses we characterized the complete genome of influenza A viruses that clearly showed different pathogenicity for mink.

## Results and discussion

The outcome of influenza A virus infection is influenced both by the virus and the infected host [11,12]. The virulence of an influenza virus isolate for a given host reflects its ability to enter a host cell, replicate within the cell and then exit and spread to new cells. Several viral gene products can contribute to the pathogenicity and virulence of the influenza A virus [13,14]. Although in most instances virulence is a multigenic trait, a single gene can also markedly affect the pathogenicity and virulence of the virus [15-18].

### Phylogenetic and sequence analysis

We sequenced the complete genome of five H10 viruses and analysed them along with all H10 viruses available in the GenBank database. Phylogenetic relationships were determined for each of the eight gene segments. The amino acid sequences of the entire genome were analysed to identify important amino acid residues associated with enhanced replication and virulence in mammalian species.

### Haemagglutinin

**Figure 1:**
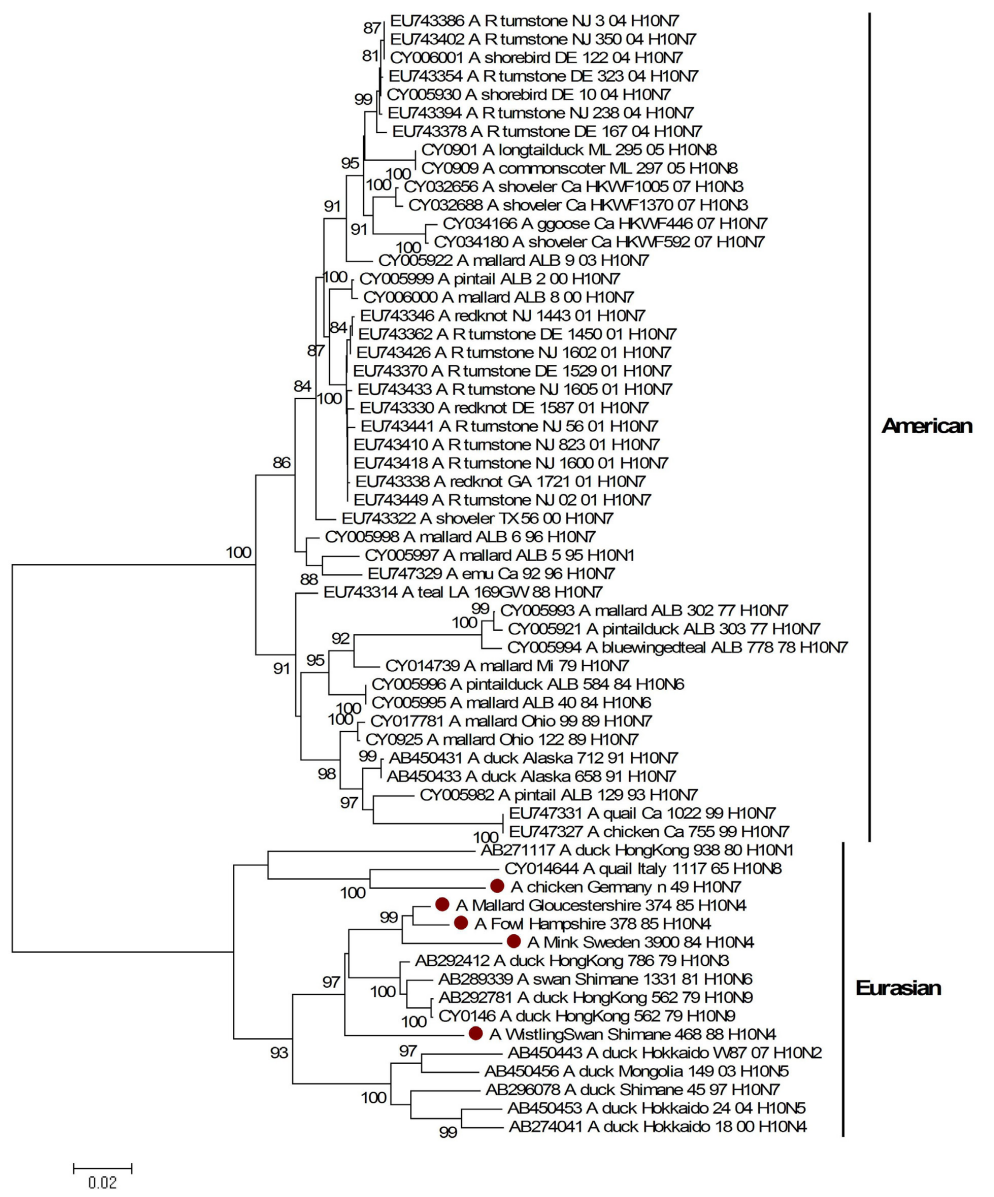
**Phylogenetic relationship between haemagglutinin genes of H10 influenza A viruses**. The protein coding region tree was generated by neighbour-joining analysis with the Tamura-Nei γ-model, using MEGA 4.0. Numbers below key nodes indicate the percentage of bootstrap values of 2000 replicates. Isolates sequenced in this study are indicated by a red dot.

Phylogenetic analysis of the HA gene revealed that all of the H10 viruses examined in this study belong to the Eurasian avian lineage of the influenza A viruses (Figure 1). Based on the limited sequence data from the Eurasian avian lineage of H10 influenza viruses that are available in GenBank, a clear determination of the genetic relationship among H10 viruses is very difficult. Furthermore, the HA gene of mink/84 clustered with mallard/85, fowl/85 and whistlingswan/88 within the Eurasian avian lineage and was distinct from the HA of chicken/49, which clusters with the early H10 Eurasian avian isolates. There is a high degree of similarity at the amino acid level of the haemagglutinin gene of the studied viruses. The HA genes of mink/84 and the concomitant wild bird isolates were 98% identical with each other and showed 95% similarity to the prototype H10 virus, chicken/49. The HA gene of H10 viruses was analysed for potential N-glycosylation sites. Our analysis indicated that all the studied H10 viruses possess five potential glycosylation sites (positions 13, 29, 236, 406 and 447) except for fowl/85, which displayed an additional glycosylation site at residue 123. Interestingly fowl/85 virus was originally isolated from a flock of sick chickens with nephropathy and visceral gout [3]. Several studies indicate that the receptor specificity of haemagglutinin plays an important role for tissue tropism and the host range of the influenza virus [19]. The amino acid composition of the receptor binding pocket of the HA protein for the H10 isolates is typical of avian influenza viruses. The H10 viruses have histidine (H), glutamate (E) and glutamine (Q) at amino acid positions 177, 184, and 216, respectively, in H10 numbering at the receptor-binding site [20], which favours binding of sialic-acid α-2,3-galactose.

The haemagglutinin cleavability and the presence of multiple basic amino acids at the HA cleavage site play a major role in influenza H5 and H7 virus transmission and virulence [21-23]. All five H10 isolates presented in this study contained the amino acid sequence *PQG*√*RGLF *at the cleavage site in the HA molecule, indicating their low pathogenicity. Nevertheless four of these H10 viruses were found to be highly pathogenic in mink. It is noteworthy that at least two H10 isolates (A/turkey/England/384/79/H10N4 and A/mandarin duck/Singapore/8058F-72/7/93/H10N5) that were reported previously by Wood and co authors, would have fulfilled the definition for highly pathogenic viruses with intravenous pathogenicity index (IVPI) values > 1.2, without having multiple basic amino acids at their haemagglutinin cleavage site [24]. This suggests that a factor other than the presence of multiple basic amino acids in the cleavage site contributed to the severity of H10 viruses in mink.

### Neuraminidase

Two different NA subtypes (N4 and N7) were associated with H10 viruses in this study. Phylogenetic analysis of the NA gene showed that all viruses belonged to the Eurasian avian lineage and within each NA subtype, the viruses clustered in the same branches. The NA protein plays an important role during the entry of the virus into the cells and in the release of viral progeny from infected cells [25,26]. The active site of the NA protein consists of 15 charged amino acids that are conserved in all influenza A viruses [27]. All of these amino acids that make up the active site (R117, D150, R151, R224, E276, R292, R369 and Y403 in N4 numbering) and the framework site (E119, R155, W178, S179, D/N198, I122, E227, H274, E277, N294 and E425) of the NA are conserved in the H10 viruses presented in this study. H10 influenza viruses have a propensity to cause clinical symptoms in humans; experimental and natural infections with H10N7 strains have clearly shown the zoonotic potential of some H10 avian influenza viruses [28,29]. In the NA protein of the analysed H10 isolates no substitutions associated with resistance to neuraminidase inhibitor drugs (oseltamivir) were observed [30].

It has been suggested that the efficiency of viral replication in terrestrial domestic poultry correlates with the length of the NA stalk and that stalk deletion has resulted in adaptation of the virus to land-based poultry [26,31]. No deletions were found in the stalk regions of the neuraminidase of the viruses sequenced in this study, indicating no adaptation for growth in terrestrial domestic poultry, this despite the fact that two of the studied viruses have been isolated from sick chickens [3,7].

### Internal genes

The ribonucleoprotein (RNP) complex of influenza virus contains four proteins that are necessary for viral replication: PB1, PB2, PA and NP. Several substitutions in the polymerase complex proteins are implicated in the virulence of the influenza viruses [32,33]. Previous studies showed that most of the host-specific markers that discriminate between human and avian influenza viruses are located in viral RNPs [34,35]. Our analysis indicates the clear avian origin of the studied viruses with two exceptions; substitution E627K in PB2 has been shown to be important for adaptation of avian viruses to replication in mammalian hosts, and interestingly, our sequence analysis showed that viruses isolated in mink and concomitant H10 viruses carry a glutamic acid (E) at position 627 which is typically found in avian viruses, while chicken/49-like human viruses have a lysine (K) substitution at position 627 of PB2. This substitution has been shown to be the main determinant of the pathogenicity of avian influenza viruses in mammalian hosts and results in increased replication of viruses in the upper respiratory tract of mice and ferrets [17,36-38]. Substitution D701N in polymerase protein PB2 has been implicated in the adaptation of H5N1 viruses to replication and high pathogenicity in mammalian hosts [35], this being the same substitution as seen in mink/84.

The recently discovered PB1-F2, a 90-amino-acid peptide translated from an alternative reading frame of the PB1 gene, induces apoptosis in infected cells [39]. The substitution N66S resulted in a more severe infection with higher virus titres and increased production of inflammatory cytokines in the lungs of infected mice [40]. None of the viruses presented in this study contained the N66S substitution. Similarity percentages for the gene segments of the RNP complex varied from 88 to 95% for the PA gene to 90-100% for the PB1-F2 at the nucleotide level.

**Figure 2:**
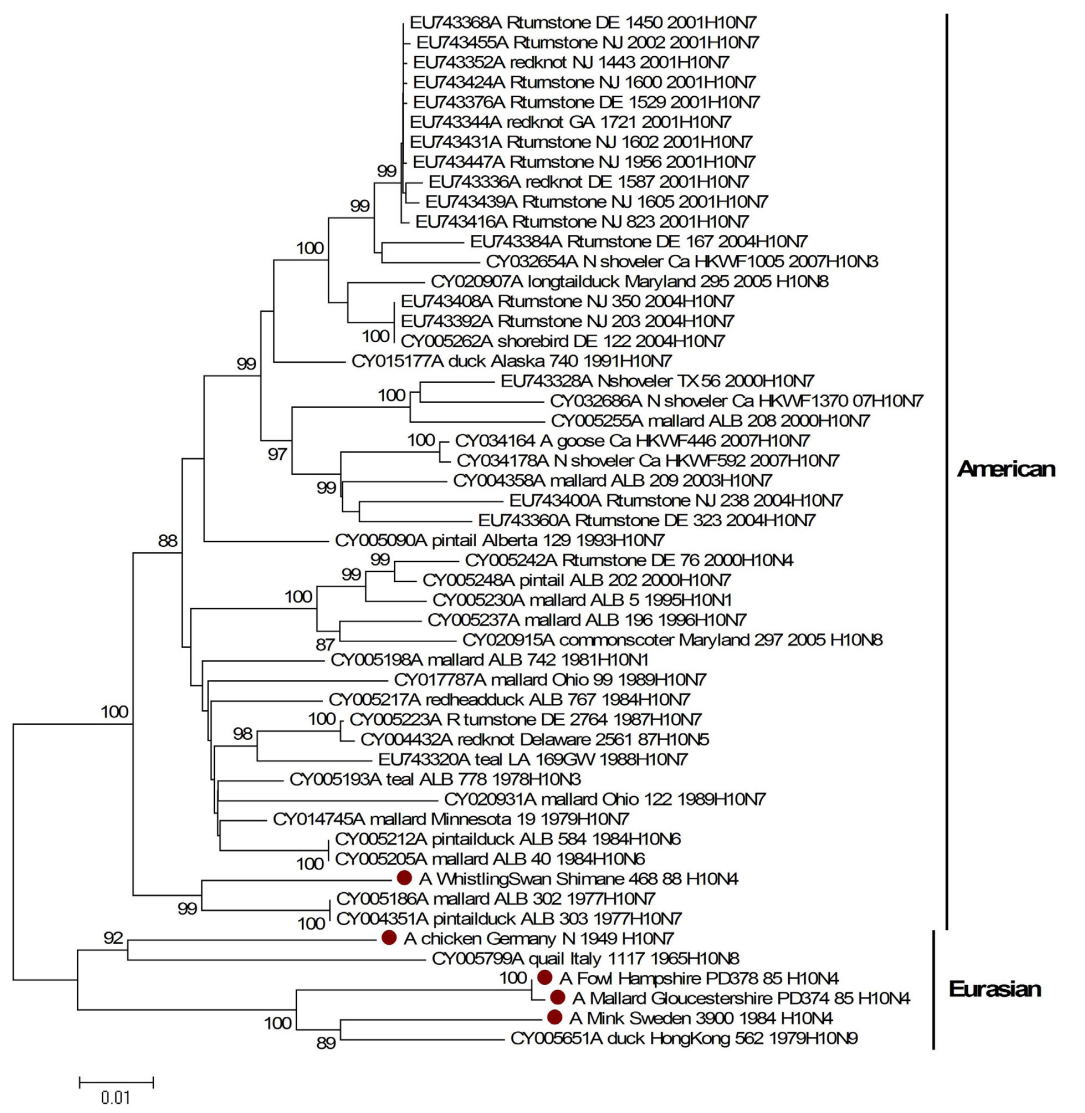
**Phylogenetic relationship between polymerase basic protein 1 genes of H10 influenza A viruses**. The protein coding region tree was generated by neighbour-joining analysis with the Tamura-Nei γ-model, using MEGA 4.0. Numbers below key nodes indicate the percentage of bootstrap values of 2000 replicates. Isolates sequenced in this study are indicated by a red dot.

Phylogenetic relationships were inferred for each of the gene segments of the RNP complex. All virus genes belong to the Eurasian avian lineage with the exception of the PB1 gene of whistlingswan/88. With regards to the PB1 gene, the whistlingswan/88 virus formed a sister branch with the main American avian lineage of H10 viruses, indicating the reassortment with genes belonging to the American avian gene pool (Figure 2).

Phylogenetic analysis showed that the M genes of the H10 viruses presented in this study are closely related to each other and all belong to the Eurasian avian lineage of the influenza A viruses. Four amino acids substitutions (L26F, V27A or T, A30T or V and S31N or R) at the M2 gene have been shown to be associated with resistance to amantadine [41], an anti-influenza drug commonly used in humans. Analysis of M2 protein amino acid sequences showed that the H10 isolates are all sensitive to amantadine.

**Figure 3:**
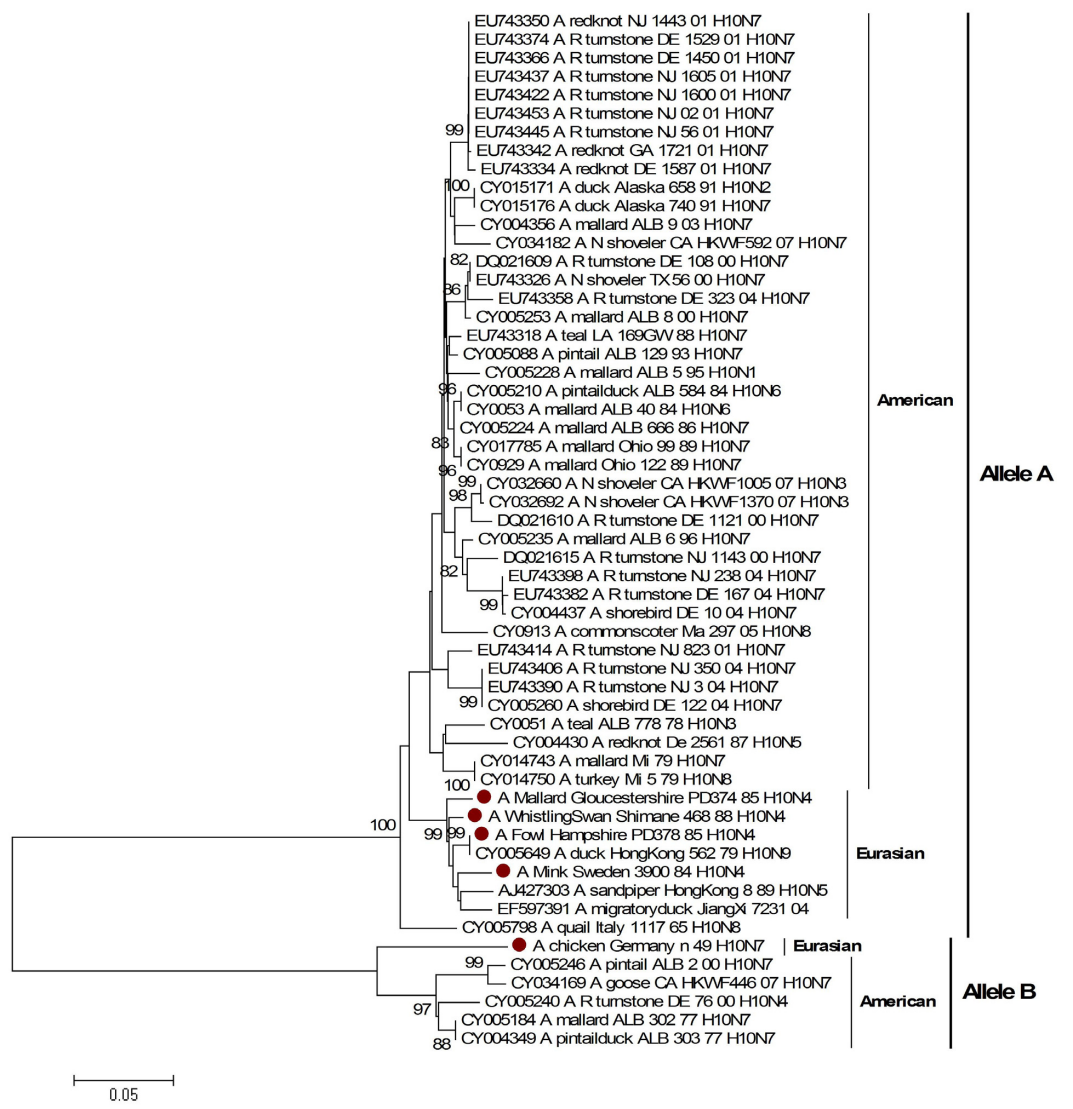
**Phylogenetic relationship between NS1 genes of H10 influenza A viruses**. The protein coding region tree was generated by neighbour-joining analysis with the Tamura-Nei γ-model, using MEGA 4.0. Numbers below key nodes indicate the percentage of bootstrap values of 2000 replicates. Isolates sequenced in this study are indicated by a red dot.

Two distinct gene pools of the non structural gene (NS), corresponding to allele A and allele B [42,43], were present among the studied H10 viruses. The NS gene of mink/84 clustered together with mallard/85, fowl/85 and whistlingswan/88 in allele A within the Eurasian avian lineage and it was clearly distinct from the NS of chicken/49, which formed a single branch as the only Eurasian avian H10 isolate among the allele B viruses (Figure 3). The NS1 genes of the H10 viruses reported in this study consisted of 890 nucleotides; there were no deletions or insertions. Nucleotide sequence identities of the NS1 gene in allele A were 95-100%, however there was 63% nucleotide identity and 69% amino acid identity between mink/84 in allele A and chicken/49 in allele B.

Several studies have identified significant amino acid motifs associated with the increased virulence of avian influenza viruses in human (D92E) and chickens (V149A) [37,44]. All the H10 viruses in our study contained 92D and 149A. Obenauer and colleagues (2006) proposed that the four C-terminal amino acid residues of the NS1 act as a PDZ binding motif that may represent a virulence determinant. PDZ domains are protein-interacting domains that are involved in a variety of cell signalling pathways. In addition, Obenauer and colleagues (2006) showed that there were typical human, avian, equine and swine motifs [45]. All the H10 viruses possessed the typical avian ESEV amino acid sequence at the C-terminal end of the NS1 protein.

The unique property of some avian H10 viruses, particularly the ability to cause severe disease in mink without prior adaptation, enabled our study. Coupled with previous experimental data and genetic studies we tried to investigate the possible influence of different genes on the virulence of these H10 avian influenza viruses in mink. Of those amino acid residues previously described as virulence factors influencing the outcome of the avian influenza virus infection in mammalian species only one was present in the H10 viruses studied here. Although Hatta *et al. *(2001) found that only a single amino acid substitution E627K of the PB2 contributes to efficient replication, effective transmission and virulence of H5N1 influenza virus in mammalian species [17], it seems that the existence of this mutation in PB2 of chicken/49 does not influence the virulence of this virus in mink. There were no differences in receptor specificity or the cleavability of the haemagglutinin proteins between H10 viruses that were shown to be of low or high pathogenicity in mink. Differences in pathogenicity and virulence between mink/84 and chicken/49 isolates could be related to clear amino acid differences in the NS1 protein.

The multifunctional influenza A NS1 protein is the most well studied of the IFN antagonistic proteins [46-48]. Mutant influenza A virus with truncated NS1 proteins are unable to replicate efficiently in normal cell cultures, and require either cells deficient in IFN-α/β production, or mice with a dysfunctional STAT 1 gene to replicate [49-52]. Several publications indicate that the NS1 protein exerts its antagonistic activity by inhibiting the dsRNA-mediated activation of protein kinase R and the activation of transcription factors NF-κB and IRF-3 [53-57]. On the other hand, others claim the primary function of NS1 RNA binding to be inhibition of the 2'-5'-oligoadenylate synthetase (OAS)/RNase L pathway [58]. Recently, interactions between NS1A and the cytoplasmic RNA helicase RIG-1 have been demonstrated, leading to inhibition of the RIG-1 mediated induction of IFN-β [59,60]. NS1 has also been related directly to virulence [37,44].

To determine whether some functional differences between the NS1 protein of mink/84 and chicken/49 isolates could explain the diversity in pathogenicity and virulence of these viruses in mink, a luciferase-based reporter system was used to test the effect of these proteins on IFN β promoter activity in poly I:C stimulated mink lung cells.

**Figure 4:**
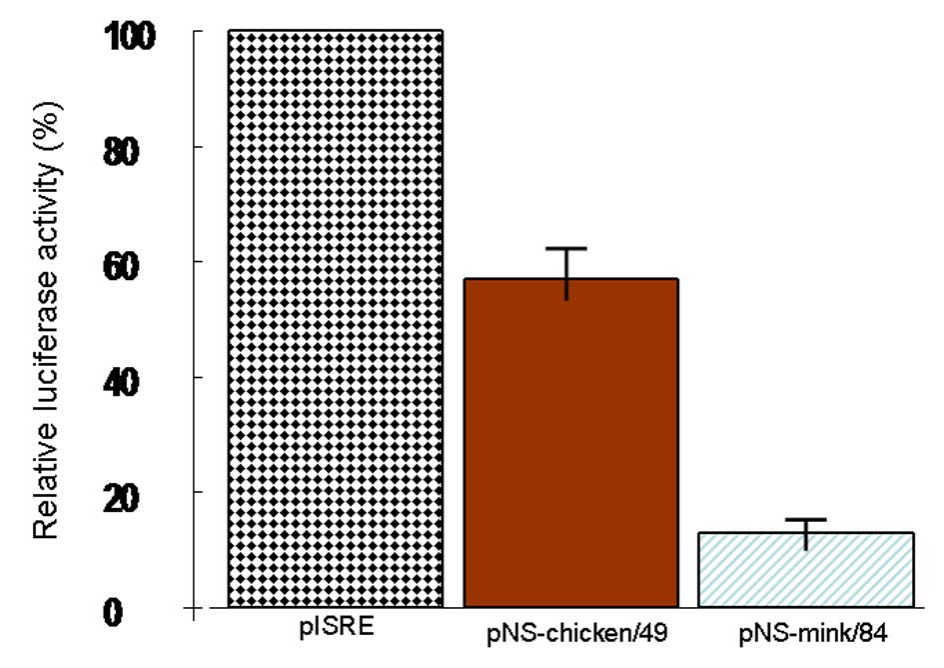
**Prevention of poly (I:C) induced activation of an IFN-β promoter in mink lung cells**. Forty-eight hours after transfection, the cells were harvested and assayed for luciferase activity. Average relative luciferase activities are reported. Data are expressed as the mean ± S.E. for the three independent experiments performed in duplicate.

Cell samples transfected with empty pcDNA3.1 vector produced the highest level of IFN promoter activity following poly I:C stimulation, and this activity level was used as a reference (set to 100%) when comparing to the rest of the samples. Samples transfected with the two NS1 proteins both exerted a negative effect on IFN promoter activity (Figure 4). The downregulation was stronger for pNS-mink/84 carrying the allele A NS gene of mink/48 virus, with an average 6.8-fold decrease (14.7%), while pNS-chicken/49 on average produced a 1.4-fold decrease (68.5%) in promoter activity compared to the reference (i.e. poly I:C stimulated cells transfected with empty pcDNA3.1 vector and reporter gene pISRE-TA-Luc). Although both proteins downregulate the IFN β promoter, the effect of the pNS-chicken/49 proteins on IFN β promoter activity was considerably weaker than that of pNS-mink/84. Production of Type I interferons (interferons α/β) represents a crucial early event in the innate immune response to viral infection [61-63].

Putative amino acid sequence analysis indicated that the sites previously been described important for the specific function of NS1 protein are similar between both NS1 of mink/84 and Chicken/49. Interestingly, one difference was noticed in the site that is considered crucial for the interaction of NS1 with the 30kDa subunit of cleavage and polyadenylation specificity factor (CPSF30). The CPSF30 is responsible for the efficient 3'-end processing of cellular pre-mRNA including IFN-b mRNA. This interaction of NS1 with CPSF30 inhibits the 3'-end processing and thus results in inhibition of cellular pre-mRNAs export from the nucleus [66,67]. Structural studies indicated that two distinct domains mediate this interaction: glutamic acid at the residue 186 (Glu186) [68] and phenylalanine and methionine at the residues 103 (Phe103) and 106 (Met106), respectively [69]. The NS1 protein of mink/84 possessed the amino acid Glu186, Phe103 and Met106, whereas the NS1 protein of Chicken/49 caries Tyr103, instead of Phe103. It was proposed earlier by Kochs and colleagues [69] that mutations at the NS1 protein and CPSF30 interaction sites dramatically changed the effect of the NS1 to control host gene expression. Thus, the effects we demonstrated here might be due to the difference in the ability of these NS1s to interfere with the cleavage and polyadenylation of IFN-β mRNA and hence suppressed IFN-β expression been observed.

## Conclusions

The NS gene of mink/84 appears to have contributed to the virulence of the virus in mink by helping the virus evade the innate immune responses. Thus, future studies are required to elucidate the mechanisms by which the NS1 protein evades innate immunity and promotes the virulence of H10 viruses.

## Methods

### Viruses

Seed stock of the influenza A viruses; A/Mink/Sweden/3900/84 (mink/84), A/fowl/Hampshire/378/85 (fowl/85), A/mallard/Gloucestershire/374/85 (mallard/85) A/whistlingswan/Shaime/88 (whistlingswan/88) and A/chicken/Germany/N/49 (chicken/49) were grown in the allantoic cavity of 9-day-old embryonated eggs and were stored at -80°C until further use.

### RNA preparation

RNA was extracted from the allantoic fluids of the infected embryonated eggs using the MagAttract Virus Mini M48 kit (Qiagen, Valencia, CA, USA) according to the manufacturer's instructions, with the Magnatrix 8000 extraction robot (Magnetic Biosolutions, Stockholm, Sweden). RNA was recovered in 70 μl of nuclease-free water and either used immediately or stored at -80°C.

### PCR

The conditions for the RT-PCRs for the different fragments were optimized to give a uniform protocol. For this purpose, the QIAGEN One-Step RT-PCR Kit (QIAGEN) was applied in a 25 μl reaction volume that comprised 5 μl of 5×buffer, 1 μl of 10 mM dNTP mix, 1 μl of each forward and reverse primer (10 pmol/μl), 1 μl of 40 U/μl RNAguard (Invitrogen, Carlsbad, CA, USA), 1 μl of enzyme, and 1 μl of template. The temperature profile was as follows: 30 minutes at 50°C for reverse transcription, 15 min at 95°C for activation of the polymerase, and then 40 cycles of 94°C for 30 sec, 52°C for 30 sec, and 72°C for 90 sec, followed by a 5 min final extension at 72°C.

### Sequence analysis

The primers for RT-PCR were segment specific but subtype universal, targeting the highly conserved regions at the 5'- and 3'-end of each segment (Table 1). PCR products were purified with the Wizard Purification Kit (Promega Corporation, Madison, WI, USA) prior to sequencing. The sequencing reaction was performed by an ABI PRISM BigDye Terminators v3.1 Cycle Sequencing Kit (Applied Biosystems, Foster City, CA, USA) according to the manufacturer's instructions. Reactions were run on a 3100 DNA analyser (Applied Biosystems). Sequencing was performed at least twice in each direction.

**Table 1:** Primers used in this study.

Gene	Primer name	Primer sequence (5'-3')
PB2	PB2-F1	TATTGGTCTCAGGGAGCGAAAGCAGGTC
	PB2-R1	TCYTCYTGTGARAAYACCAT
	PB2-F2	TAYGARGARTTCACAATGGT
	PB2-R2	ATATGGTCTCGTATTAGTAGAAACAAGGTCCTTT
PB1	PB1-F1	TATTCGTCTCAGGGAGCGAAAGCAGGCA
	PB1-R1	TTRAACATGCCCATCATCAT
	PB1-F2	ARATACCNGCAGARATGCT
	PB1-R2	ATATCGTCTCGTATTAGTAGAAACAAGGCATTT
PA	PA-F1	TATTCGTCTCAGGGAGCGAAAGCAGGTAC
	PA-R1	TNGTYCTRCAYTTGCTTATCAT
	PA-F2	CATTGAGGGCAAGCTTTC
	PA-R2	ATATCGTCTCGTATTAGTAGAAACAAGGTACTT
HA	HA-F1	GCAAAGCAGGGGTCACAATGTCA
	HAR1	TCTGAATCAGCCATGTCAATTGT
	HA-F2	GATTTCCATTGGACGATGGTACAACCA
	HA-R2	GGGTGTTTTTAACTAAATACAGATTGTGC
NP	NP-1F	AGCRAAAGCAGGGTDKATA
	NP-1R	CYARTTGACTYTTRTGTGCTGG
	NP-2F	TAYGACTTTGARAGAGAAGG
	NP-2R	AGTAGAAACAAGGGTATTTT
NA	N4-F	AGCAAAAGCAGGAGTTTCATAATGA
	N4-R	CATGGCCCGATGGCGCTCTGTTG
	N7-F	GTGATCGAGAATGAATCCAAATCAGA
	N7-R	GCATTTTACGAAAAGTATTGGATTTG
M	M-F	AGCRAAAGCAGKTAG
	M-R	AGTAGAAACAAGGTARKTTTT
NS	NS-F	CAAAAACATAATGGATYCCAACACK
	NS-R	ATTAAATAAGCTGAAAMGAGA A

Sequence assembly, multiple alignments and alignment trimming, nucleotide sequence translation into protein sequence and processing were performed with the BioEdit software v.7.0.4.1 [70] with an engine based on the Clustal W algorithm [71]. The phylogenetic analysis, based on complete gene nucleotide sequences, was conducted using Molecular Evolutionary Genetics Analysis (*MEGA*, version 4.0) software [72] using neighbour-joining tree inference analysis with the Tamura-Nei γ-model, with 2000 bootstrap replications to assign confidence levels to branches. Identification of potential glycosylation sites was done with the PPSearch programme, available at http://www.ebi.ac.uk/ppsearch.

### IFN β promoter luciferase assay

To determine some functional aspects of the NS1 protein of A/mink/Sweden/3900/84 and A/chicken/Germany/N/49 isolates, a luciferase-based reporter system was used to test the effect of these proteins on interferon β (IFN-β) promoter activity in poly I:C stimulated mink lung cells (MiLu-Cells).

### Construction of expression plasmids

The NS1 genes of influenza A virus strains A/mink/Sweden/3900/84 and A/chicken/Germany/N/49 were amplified by using the primers NS1Kpn 5' (5'-ATTCGGTACCAGCAAAAGCAGGGTGACAAAG-3') and NS1XhoI 3' (5'-TACCCTCGAGGCTATCAAACTTCTGACTCAATTGTTCTC-3'). The 690 bp PCR products were digested with *Kpn *and *Xho*I and cloned between the *Kpn *and *Xho*I sites of the mammalian expression vector pcDNA3.1 (Invitrogen), creating pNS-mink/84 and pNS-chicken/49 plasmid respectively. The integrity of the PCR products was confirmed by sequencing.

### Cell culture and transfection experiments

The mink lung cells were obtained from the Swedish National Veterinary Institute. The mink lung cells were routinely grown in Dulbecco's modified Eagle's medium (DMEM) supplemented with 5% FCS in a well humidified atmosphere of 5% CO_2 _at 37°C.

The transcriptional activity of the IFN-β promoter was assayed in mink lung cells. Cells were co-transfected with a plasmid containing either the NS gene of mink/84 or chicken/49 together with a reporter plasmid driving expression of *Firefly *luciferase (pISRE-TA-Luc) (Invitrogen) under control of the interferon-stimulated response element (ISRE), the IFN-β promoter. The pRen-Luc plasmid containing the *Renilla *luciferase gene (Invitrogen) was used as the luciferase control. The luciferase activities of the reporter gene were standardized by *Renilla *luciferase activity. NS1 activity was expressed as folds of luciferase activity.

Transfection of the plasmids was conducted with FuGENE 6 transfection reagent (Roche Molecular Biochemicals, Indianapolis, IN, USA) in six-well plates according to the manufacturer's instructions. Initial experiments were conducted to increase the efficiency of the transfection protocol. The day before transfection, MiLu-cells were collected, and seeded into six-well plates at 1×10^5 ^cells per well in order to achieve 70%-80% confluence on the day of transfection. Each transfection group consisted of six wells in which three were poly I:C stimulated and three were mock treated. Stimulation of the cells with the poly I:C was performed 24 hours after transfection of pcDNA3.1/NS1 plasmid, through the addition of 5 μg/ml poly I:C mixed in 100 μl DMEM without serum. Twenty-four hours later, the cells were harvested according to the protocol for the luciferase assay kit (Stratagene, Heidelberg, Germany), using 300 μl lysis buffer for each well. Samples were kept on ice and centrifuged for 2 min at 14,000 × g for removal of the cell debris prior to measurement of the luciferase activity. Luciferase activities were measured using 20 μl of each sample according to the manufacturer's protocol.

### Nucleotide sequence accession numbers

The nucleotide sequence data obtained in this study has been submitted to the GenBank database and is available under accession numbers; GQ176105-GQ176144.

## Competing interests

The authors declare that they have no competing interests.

## Authors' contributions

SZ conceived and designed the study, organized protocol developments, carried out PCR and sequencing reactions, performed sequence analyses, alignments, phylogenies, interpretation of data and wrote the manuscript. GM took part in development of amplification protocols, carried out PCR and sequencing reactions, performed sequence analyses, contributed to and revised the manuscript. IK, organized protocol developments, contributed to the interpretation of the findings and revised the manuscript. SB contributed to conception, interpretation of data, and revised the manuscript. MB additionally contributed to the study design contributed to conception, interpretation of data and revised the manuscript. All authors' have read and approved the final manuscript.
